# Bibliographical

**Published:** 1881-05

**Authors:** 


### BIBLIOGRAPHICAL.

The proceedings of the "New Jersey State Dental Society," for the years 1878–9–80 have just come to hand. It is a neatly gotten up bound volume of 209 pages, filled with interesting and instructive matter in the form of minutes of the meetings, essays and discussions; in these will be found many fresh and new thoughts and suggestions that will well repay a thorough perusal—yes study.

All such publications should be in the hands of, and read by every progressive member of the profession.

This volume can be obtained of either Dr. Chas. A. Meeker or Dr. John C. Hanks, Newark, N. J. *Send for it.*

---

The volume of "Transactions of the Ohio State Dental Society." Session of 1880 has just made its appearance. It contains 72 pages, in which is discussed important professional subjects of eminently a practical nature, and interesting to every dentist.

The volume may be obtained of the publishers, Messrs. Ransom & Randolph, Toledo, O., or of the Editor of the REGISTER, Cincinnati, or of Dr. W. H. Sillito, Xenia, O. Send a three cent stamp.

---

The volume of "Transactions of the Twentieth Annual Meeting of the American Dental Association," is at hand. The work is well gotten up in every respect. The fact that it was published by the Trustees of S. S. White, is a sufficient guarantee in that direction.

This is the most important and valuable volume of this character published by any society in the country. It is the production of more of the leading minds in the profession than can be brought together in any State or local society; this fact adds great value to the work.

It ought, at least, to be in the hands of every member of a dental society in the country, for it gives a better idea of the extent and results of association effort than can be gained in any other way.

Indeed, all should have it.

It may be obtained of the publishers, or of Dr. Geo. H. Cushing, chairman of the publication committee. It is sold at about the cost of publication.

